# Intensive Ways of Producing Carbonate Curing Building Materials Based on Lime Secondary Raw Materials

**DOI:** 10.3390/ma13102304

**Published:** 2020-05-16

**Authors:** Nikolai Lyubomirskiy, Aleksandr Bakhtin, Stanisław Fic, Małgorzata Szafraniec, Tamara Bakhtinа

**Affiliations:** 1 Department of Civil Engineering and Materials Science, Faculty of Architecture and Civil Engineering, Academy of Civil Engineering and Architecture, V.I. Vernadsky Crimean Federal University, Prospekt Vernadskogo 4, 295007 Simferopol, Republic of Crimea; niklub.ua@gmail.com (N.L); aleserba@gmail.com (A.B.); t.bakhtina83@gmail.com (T.B.); 2 Faculty of Civil Engineering and Architecture, Lublin University of Technology, ul. Nadbystrzycka 40, 20-618 Lublin, Poland; s.fic@pollub.pl

**Keywords:** forced carbonate hardening, secondary raw materials, soda production wastes, lime dust, Ca(OH)_2_, CaCO_3_, CO_2_, carbonation chamber

## Abstract

The article is dedicated to the research and development of intensive methods for curing products by capturing and binding CO_2_. It aims to improve and increase the productivity of technologies for the production of artificially carbonated building materials and products. Soda production wastes, limestone dust and finely dispersed limestone dust were used as the research objects. Secondary raw materials have been investigated using modern methods of phase composition and granulometry test. Intensive methods of production of accelerated carbonation of systems consisting of soda wastes were tested using multi-parameter optimization methods. The effects of recycled lime materials on the strength and hydrophysical properties of the obtained material were determined. The secondary raw materials effect depended on the composition of the raw mixture, molding conditions, CO_2_ concentration applied to the carbonate curing chamber, and the duration of exposure to environments with high CO_2_ content. It was found that the most effective way of providing accelerated carbonation curing of construction materials and products is a combined carbonation method, combining the principles of dynamic and static methods. It was concluded that the optimal CO_2_ concentration in the gas-air mixtures used for carbonate curing is 30%–40%.

## 1. Introduction

The global environmental problems of climate change as a result of human impacts on nature have been firmly established as the most internationally discussed issues (Conference of Parties (COPs) in Warsaw (COP-19 in 2013), in Lima (COP-20 in 2014), in Paris (COP-21 in 2015), and most recently in Marrakech (COP-22 in November of 2016). The increasing concentration of greenhouse gases in the atmosphere at the current stage of economic development requires the development of new products and production methods characterized by lower CO_2_ emissions than traditional approaches. 

One of the branches of industry that emits a significant amount of anthropogenic CO_2_ into the atmosphere is the construction materials industry, which is in second place after the energy sector in terms of global CO_2_ emissions. Thus, the production of Portland cement and other binders alone accounts for up to 8% of the world’s anthropogenic CO_2_ emissions [1,2,3,4]. In this sense, CO_2_ sequestration is one of the most important technical challenges of our time [5].

It is noteworthy that at the same time, it is the construction industry that has significant potential in terms of creating technologies for the production of various materials and products that use carbon dioxide in their manufacturing process. It is shown in [6,7] that CO_2_ can be used both as an effective additive of cement concrete curing accelerator and as the main raw material component [8,9,10]. Recently, there has been a steady and growing interest in various alternative binders and materials with binder properties, containing minerals that can interact with CO_2_ and bind it into stable compounds.

A significant reserve of raw materials in the production of building materials and products hardening due to carbon dioxide are various wastes and industrial waste products, which at certain technological processing can exhibit bindering properties and enter into chemical interaction with carbon dioxide, forming an artificial stone. As a result of numerous research studies [11,12,13,14,15], a number of kinds of the secondary raw materials possessing the essential potential for СО_2_ binding have been revealed, scientific and technological bases for introduction of the received results in the industry have been developed [13,14,15,16], and moreover, pilot batches of the building products hardened in the environment of the raised concentration of СО_2_ have been issued [13,14,15,16].

One of the promising secondary raw materials for the production of carbonate hardening building materials is the waste generated in the production of soda ash. The chemical process of soda ash production using ammonia technology is based on five reactions, one of which is the production of calcium oxide and carbon dioxide as a result of carbonate rock cooking in lime kilns [17]. As a result of cleaning the carbon dioxide for its further use, a by-product is obtained, the so-called lime dust, which has low activity and does not find a proper further application. In several of the conducted scientific research studies, the directions for the use of by-products of soda manufacturing for the production of building materials and products are defined [18,19], including low-active lime. However, in practice, it does not occur, and does not allow one to leave the problem of accumulation of the given by-product, in dumps and in its negative influence on the environment. The research carried out in [20] has established the possibility of using lime dust for production of building materials by the method of forced carbonation, which has high physical and mechanical characteristics. The filler in these materials was finely dispersed limestone, which is also a by-product of the sorting of limestone crushed to produce lime and carbon dioxide. The analysis of utilization directions of soda production wastes in construction materials of forced carbonate hardening has shown that carbonate technology allows one to fully utilize all formed solid by-products, as well as carbon dioxide emitted into the atmosphere, and does not require special preparation, so one can directly use it without any cleaning. Such a solution is inexpensive, quite simple from the technical and technological point of view and allows one to significantly reduce the formation of waste during the production of soda, and due to the reduction of CO_2_ emissions into the atmosphere to significantly increase the environmental friendliness of the technology, it is transferred to the "low-carbon" class.

Forced carbonation is an active curing process and allows the production of materials with high physical and mechanical characteristics within a short time period (1–3 h) [20,21,22]. However, issues related to the increase of productivity of technologies of the manufacturing of artificially carbonated building materials and products at the expense of applications of more intensive methods of their forced carbonation and regularities of formation of the properties of received materials thus cause certain scientific and practical interest.

In this regard, the main idea of the performed research work is to research and develop intensive methods of forced carbonation of the systems consisting of soda production wastes—lime dust and finely dispersed limestone, and their influence on the formation of the basic properties corresponding for structural building materials and products, depending on the various formulation and technological factors. For this purpose, a series of experiments have been conducted, the main results of which are presented in this article.

## 2. Materials and Methods

### 2.1. Materials 

The materials used for scientific research were lime dust mechanically precipitated in cyclones and bag filters of mine lime kilns that calcin limestone. Finely dispersed marble limestone with fraction up to 5 mm, formed in the process of crushing and sorting the initial rock into a working fraction sent to the shaft kiln for cooking, was also used.

The activity of lime dust, determined by standard methods of lime testing, was 45%, humidity 0.6% wt., temperature and quenching time are 41 °С and 47 min, respectively.

The phase composition was determined using Perkin Elmer’s STA 8000 (PerkinElmer Inc., Waltham, Massachusetts, USA) high-temperature synchronous analyzer (USA), in the temperature range of 30–1000 °С, at a heating rate of 10 °С/min in a nitrogen environment. The particle size of the investigated materials was determined on the laser analyzer HORIBA LA-960 (HORIBA, Ltd. Kyoto, Japan).

The lime dust is factory stored and stored in bulk in an open fenced area. It is natural to believe that under such storage conditions, a portion of the free СаО contained in it is extinguished and transferred to Ca(OH)_2_ and CaCO_3_. Quantitative changes in the phase composition of lime dust depending on the conditions of its pre-preparation were investigated using synchronous Thermogravimetric and Differential thermal analysis (TG-DTA) analysis under a dynamic nitrogen atmosphere. Samples were tested in their natural state and after quenching under different conditions, in order to determine the presence of calcinated CaO particles. Samples were dried before testing. Changes in the content of the main phases in lime dust under various conditions of its preliminary preparation are presented in Table 1.

The analysis of experimental data on changes in the mineralogical composition of lime dust depending on the conditions of its preparation show that in its natural state lime dust is in a hydrated state and contains about 40% Ca(OH)_2_. Equivalent to CaO, this is 30.5%. TG-curves of freshly slaked lime dust samples show that the Ca(OH)_2_ content is increasing, however, it can be seen from the CaO content that there is still a significant amount of free calcinated CaO particles in the composition. After quenching and the additional maintenance of products of quenching of lime dust during 120 and 420 min, and also after boiling and maintenance during 420 min, according to the data of TG-DTA curves, the quantity of Ca(OH)_2_ with increase in time of maintenance increases to 55.1%, and after boiling, increases up to 59.5%. Based on the results of the thermal analysis, the amount of calcinated CaO particles in the lime dust is about 7%. According to the analysis, lime dust is found to be one-third composed of calcium carbonate—uncalcinated limestone particles. In addition, lime dust in its composition contains admixtures of silica (SiO_2_) and uncalcinated hard coal, manifested on thermograms in the form of exothermic effects in the temperature range of 350–400 °C.

**Figure 1:**
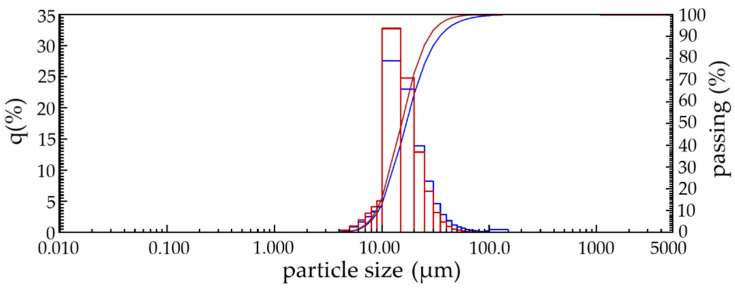
Dispersion composition and curve of total lime dust particles: **−−−** before quenching; **−−−** after quenching.

Determination of the size of lime dust particles in the quick and slake state (Figure 1) by laser diffraction showed that its particle size composition is 8 to 30 microns, and after quenching the resulting material contains mainly particles with sizes from 6 to 20 µm. The average geometric particle size of lime dust before and after quenching is 16.7 and 12.9 µm, respectively. Considering such a small size of particles of lime dust, it can be argued that the destructive effect of calcinated particles on the properties of products made on its basis will be minimal.

As finely dispersed limestone in the work used by-products of crushing and sorting of limestone Balaklava deposit (Crimea) fraction to 5 mm, formed during the crushing and sorting of the original rock to the working fraction sent to the mine kiln for cooking. The main properties and content of the main rock-forming chemical compound calcium carbonate limestone are presented in Table 2.

**Figure 2:**
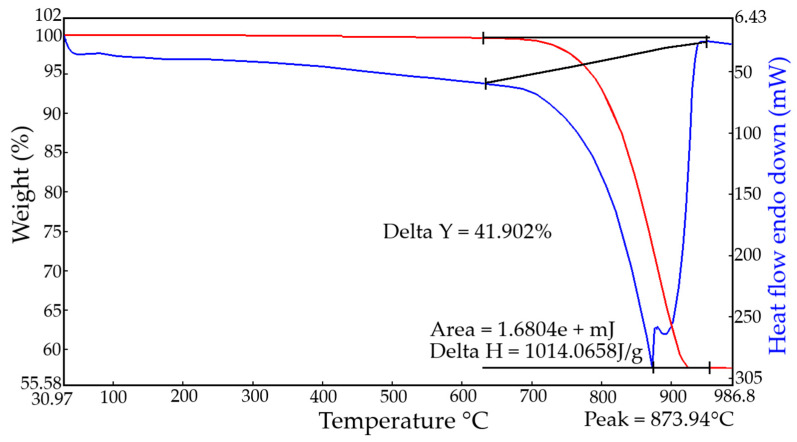
Thermogram of Balaklava deposit limestone sample.

Limestone from the Balaklava deposit belongs to nummulite partially recrystallized (metamorphosed) limestone and is a sedimentary dense and sufficiently strong rock. The thermogram of limestone (Figure 2) has one endothermic effect, corresponding to the decomposition of calcium carbonate. The endothermic effect has two peaks, which may indicate that the studied calcium carbonate is mainly represented by calcite and possibly contains minor impurities of dolomite [23,24].

### 2.2. Methods

Liquid high-pressure carbon dioxide in cylinders was used as a source of carbon dioxide in laboratory studies.

Research studies were carried out on the cylinder samples made by a method of semi-dry pressing of the molding mixtures, consisting of thoroughly mixed in a speed mixer of lime dust and finely dispersed limestone, passed through a sieve with the size of mesh 1.25 mm. The water content of the molding mixtures was 10% wt. These values are justified by the results presented in [21,22,25]. The compositions of the molding mixtures are determined by the experimental conditions and are presented below. The 30 mm diameter testing cylinder samples were formed by pressing the raw mix in metal molds on a hydraulic press, with a simulation of double-sided counter-pressing. The diameter/height ratio was maintained within 1.

The forced carbonation of testing samples was carried out in the carbonation unit with automatic control and maintenance of the required concentration of CO_2_ in it [26]. The unit was additionally equipped with a buffer tank to prepare and maintain a constant concentration of CO_2_ gas and air mixtures.

Research studies of complex influence of the composition of a raw mix, factors of reception, and also modes of the accelerated carbonation of pre-production of testing samples were spent by means of statistical methods of mathematical designing of the experiment [27,28]. Central composite rotatable design (CCRD) was adopted. A central composite rotatable design of the experiment—design points are located on three spheres: central points (zero-radius sphere), cube points and "star points". Rotatable designs allow getting the coefficients of models that predict the output value of the object with the same accuracy in all directions at the same distance from the center of the design. The obtained experimental data were processed using the STATISTICA 12 software package (StatSoft, Inc., Tulsa, USA). As a result of processing the testing data, the experimental-statistical (ES) models of each investigated parameter are formed, representing the equations of the second-order of the next kind:

(1) $$Y=b_{0}+\sum_{i=1}^{n}b_{i}z_{i}+\sum_{i;j=1}^{n}b_{ij}z_{i}z_{j}+\sum_{i=1}^{n}b_{ii}{z_{i}}^{2},$$

where b_0_, b_i_, b_ij_, b_ii_ is correlation coefficients determined as a result of mathematical and statistical processing of experimental data; z_i_, z_j_ is the value of variable formulation and technological factors.

The significance of ES model correlation coefficients was determined with the help of the Student’s criterion; the second-order equation checked the adequacy of the object description by the Fisher’s F-criterion.

Two four-factor experiments differing only in the method of forced carbonation—dynamic (cyclic) and static—were implemented.

The principle of the applied dynamic method of carbonation implied accelerated carbonation of the lime binder within several cycles. One carbonation cycle included several consecutive operations: vacuumization of the sample chamber, holding under conditions of rarefaction, carbon dioxide supply to the carbonation chamber, holding the samples in CO_2_ environment of a certain concentration. The rarefaction in the chamber was 5000 Pa, the prepared in advance to the required concentration gas and air mixture of CO_2_ was fed into the chamber from a buffer tank. The gas, seeping through the pores of the product, returns to the buffer tank, where it is brought to the desired concentration by feeding the required amount of CO_2_. The duration of one cycle was 180 s.

The statistical method of carbonation was based on storing testing samples in carbon dioxide gas and air environment, without creating the resolution in the carbonation chamber. Duration of carbonation of testing samples corresponded to the duration of carbonation under the dynamic method.

The choice of factors in the experiment was made, taking into account the conclusions obtained in the analysis of the structure formation and properties of artificial stone based on lime carbonate hardening [21,22,25]. Taking into account the influence of many factors influencing the lime carbonation process and the formation of material properties on its basis in this experiment varied: the concentration of CO_2_ (z_1_), the forming pressure of pressing the testing cylinder samples (z_2_), the content of lime dust in the lime-lime composition equivalent to Ca(OH)_2_ (z_3_) and, directly, the modes of forced carbonation, the number of cycles and time of exposure to CO_2_ on the testing samples (z_4_). The designing conditions for the experiment are presented in Table 3.

Main parameters of testing samples were compressive strength (R_c_), average density (*ρ*_o_), water resistance (K_S_), water absorption by mass (W_m_) and carbonated layer thickness (δ).

Compressive strength, average density and water absorption by weight were determined using standard methods for building materials. The resistance of testing samples, i.e. the ability of the material to maintain its operating properties under prolonged exposure to water, was evaluated by the so-called softening factor, K_S_, which is the ratio of compressive strength of the sample in a water-saturated state, R^w^ to the compressive strength of the material in a dry state, R^d^ by the formula:

(2) $$K_{s}=\frac{R^{w}}{R^{d}},$$

Water-resistant materials are those with K_S_ above 0.8.

The thickness of the carbonated layer, δ, was determined by spraying, on the surface, a 1% alcohol solution of phenolphthalein to the testing sample. 

Testing samples were dried to zero humidity in a drying cabinet at 80 °C, before testing. 

## 3. Results

Experimental data on the properties of testing samples carbonated by dynamic (1) and static (2) methods are presented in Table 4.

The correlation coefficients of ES models, obtained as a result of statistical processing of testing data, are presented in Table 5.

The estimation of the influence of separate technological factors shows that the importance of their influence on the formation of properties of materials based on lime carbonation hardening depending on the chosen method of production of artificial carbonation is different. So, if the dynamic method of carbonation (model 1), the most influencing factors can be called the factors for obtaining the testing samples, i.e. pressure pressing (z_2_) and lime dust content (z_3_), then in the static method (model 2), along with the main factor z_2_, the factors responsible directly for the process of carbonation curing, such as the concentration of CO_2_ (z_1_) and carbonation time (z_4_), become more decisive.

Graphical processing of the received ES models has allowed receiving a set of surfaces of the response of change of investigated parameters from varied factors at their joint influence in various combinations (Figure 3, Figure 4, Figure 5, Figure 6, Figure 7, Figure 8, Figure 9, Figure 10, Figure 11 and Figure 12).

**Figure 3:**
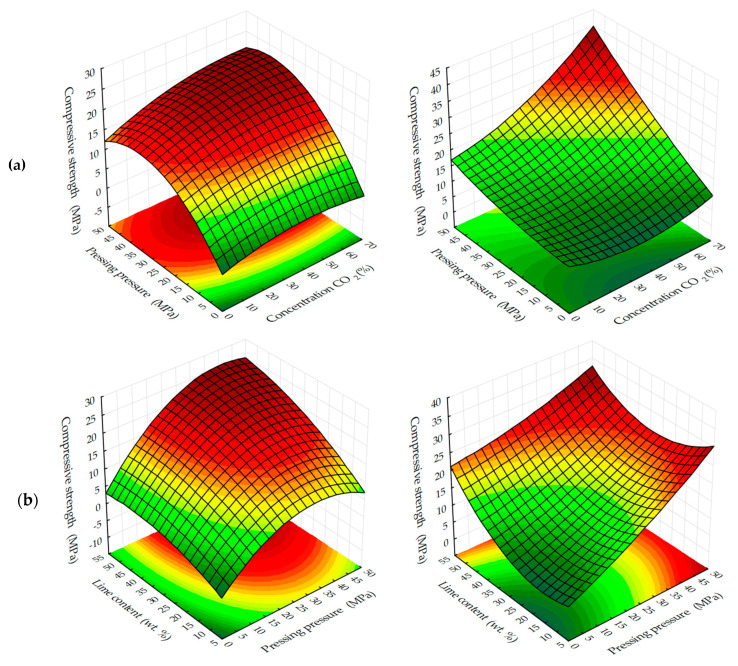

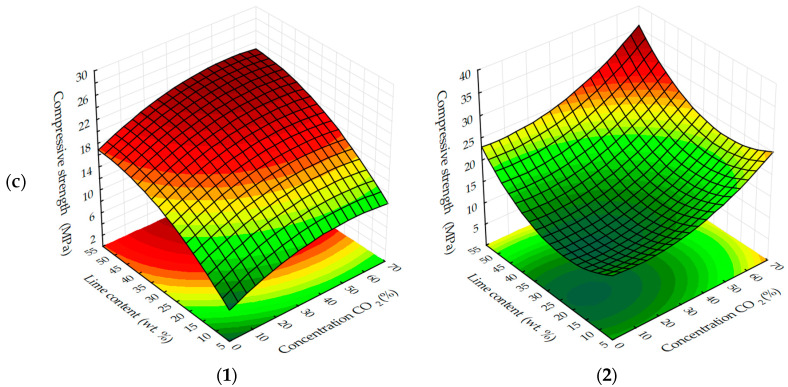
Compressive strength response surfaces of testing samples carbonated by dynamic (**1**) and static (**2**) methods, depending on: (**a**) pressing pressure; (**b**) lime dust content; (**c**) CO_2_ concentration (at zero level of variation of other factors).

**Figure 4:**
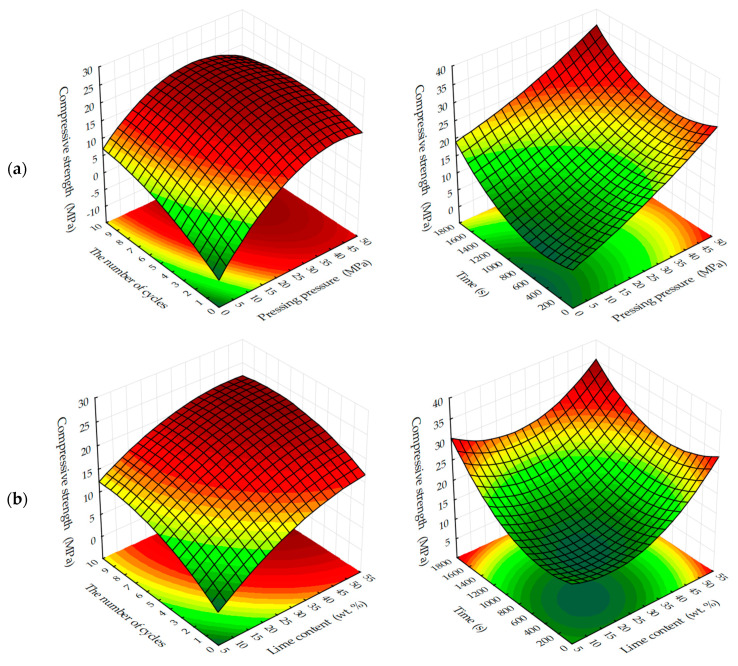

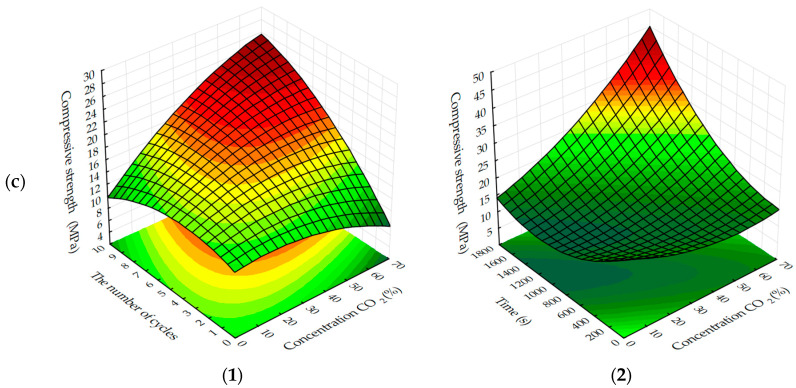
Compressive strength response surfaces of testing samples carbonated by dynamic (**1**) and static (**2**) methods depending on a number of cycles/time of carbonation and: (**a**) pressing pressure; (**b**) lime dust content; (**c**) CO_2_ concentration (at zero level of variation of other factors).

Analyzing the experimentally calculated results of the formation of compressive strength of testing samples depending on the investigated factors (Figure 3 and Figure 4), it is necessary to note the different character of response surfaces. Thus, in the dynamic method of the carbonation of the cylinder, samples of the response surface have a dome-shaped, convex-like parabola, which has the area of points reaching the maximum values of Rс, and in the static method of carbonation of the response surface, on the contrary, somewhat concave, reminiscent of a graph of the degree function, by which the compressive strength increases with the increase of variable factors. Such characteristic differences in the response surfaces indicate that the carbonation process is more intensive in a dynamic method than in a static method, however, the estimation of absolute values of the index Rс shows that in a static method, the compression strength of carbonated cylinder sample s with prolonged maintenance in an environment with a high concentration of CO_2_ is significantly higher and tends to further increase.

**Figure 5:**
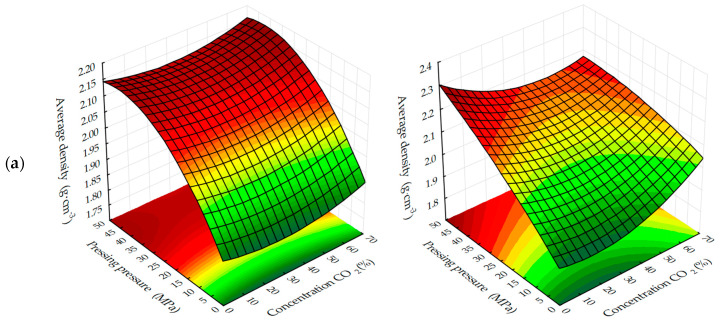

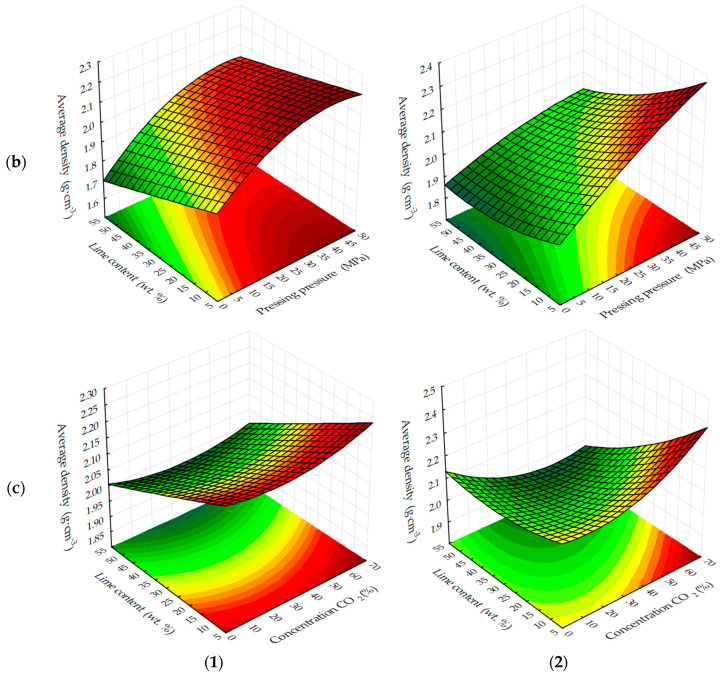
Response surfaces of changes in the average density of testing samples carbonated by dynamic (**1**) and static (**2**) methods, depending on: (**a**) the forming pressure of pressing and concentration of CO_2_; (**b**) the content of lime dust and forming pressure pressing; (**c**) the content of lime dust and concentration of CO_2_ (at zero level of variation of other factors).

**Figure 6:**
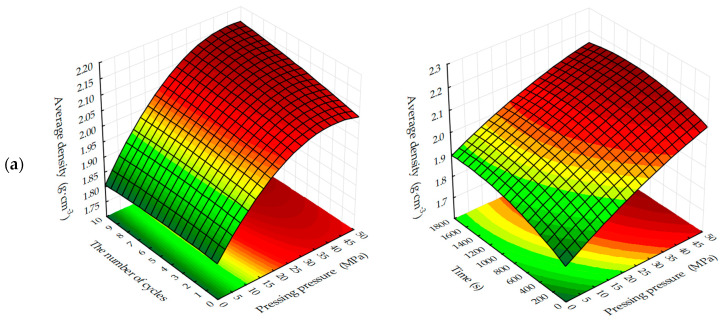

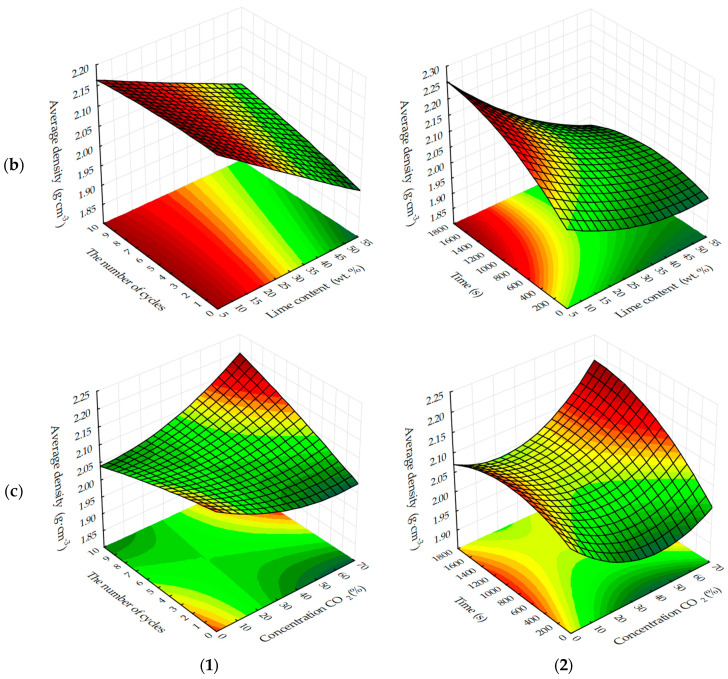
Response surfaces to changes in the average density of testing samples carbonated by dynamic (**1**) and static (**2**) methods depending on the number of cycles/carbonation time and: (**a**) pressing pressure; (**b**) lime dust content; (**c**) CO_2_ concentration (at zero level of variation of other factors).

The response surfaces of changes in the average density of carbonated samples (Figure 5 and Figure 6) show that, regardless of the methods of carbonation, the index *ρ*_o_ of testing samples increases with a decrease in the amount of lime dust and an increase in the forming pressure of pressing, due to the formation of a more dense structure of the material at the stage of production of cylinder samples and, practically, does not change with the variation of CO_2_ concentration. Average density, as well as the compressive strength of testing samples, increases more intensively with the dynamic method of carbonation, but the absolute values of the index ρ_o_, with an increase in the duration of exposure of samples in the carbon dioxide gas and air environment, are higher for the samples, the carbonation of which was carried out in a static mode.

**Figure 7:**
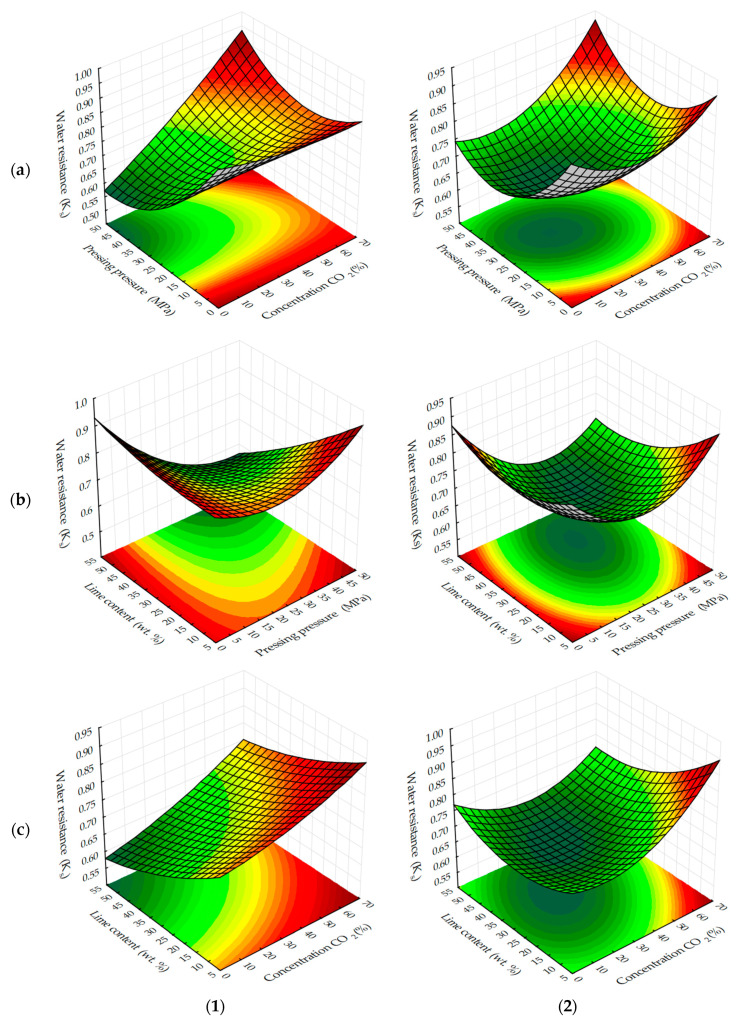
Response surfaces to changes in the water-resistance of testing samples carbonated by dynamic (**1**) and static (**2**) methods, depending on: (**a**) pressing pressure and CO_2_ concentration; (**b**) the lime dust content and pressing pressure; (**c**) the lime dust content and CO_2_ concentration (at zero level of variation of other factors).

**Figure 8:**
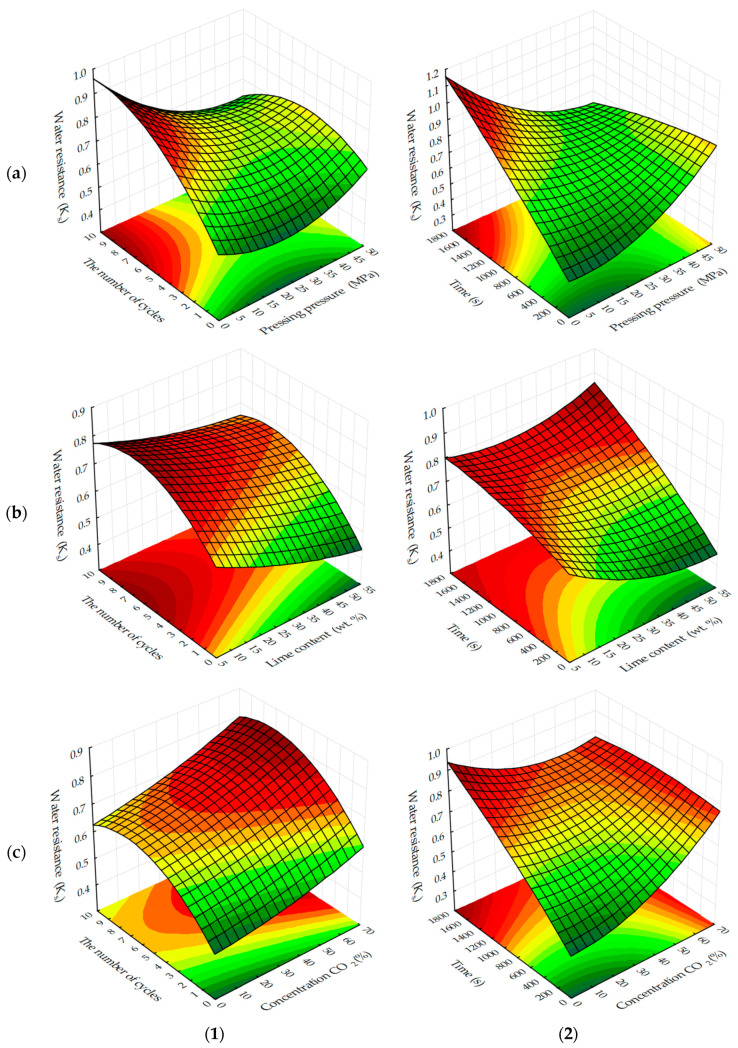
Response surfaces to changes in the water-resistance of testing samples carbonated by dynamic (**1**) and static (**2**) methods, depending on the number of cycles/time of carbonation and: (**a**) pressing pressure; (**b**) lime dust content; (**c**) CO_2_ concentration (at zero level of variation of other factors).

The areas in the graphs where the testing samples reach values at which they can be considered water-resistant, i.e. K_S_ ≥ 0.8, are not significant (Figure 7 and Figure 8), regardless of carbonation methods.

Water-resistance is mainly provided by carbonated cylinder samples obtained at forming pressures up to 10 MPa, as well as samples containing a minimum amount of lime dust (Ca(OH)_2_), in the composition of forming mixtures. Most likely, water resistance depends on the degree of carbonation contained in them Ca(OH)_2_, which under the above conditions and under accepted time factors, is characterized by maximum K_S_ values.

In view of the above, it is also logical that as the duration of carbonation (Figure 8) increases, so does the water resistance of the testing samples. Moreover, a more stable increase of this index is observed in samples obtained in the static mode of forced carbonation. With the dynamic carbonization method, K_S_ values corresponding to water-resistant materials are achieved earlier. For example, at the minimum content of lime dust (see Figure 8c), a material with K_S_ ≥ 0.8 can be obtained on the fifth cycle, and with a static method of carbonation, the same index is achieved, approximately 1400, with the holding in an environment of CO_2_, which corresponds to eight cycles, with a dynamic method of forced carbonation. Based on the results obtained, it can be argued that the water-resistance of carbonated materials depends on the degree of carbonation of the lime component, which in turn, depends on the initial structure density and carbonation time of samples based on lime dust and finely dispersed limestone.

**Figure 9:**
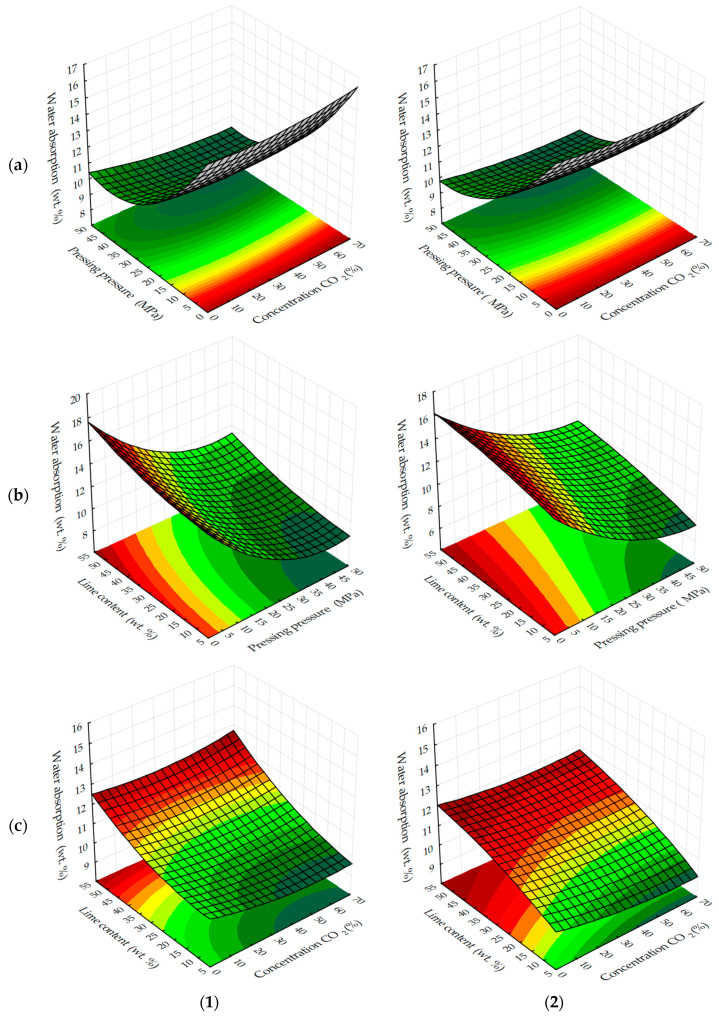
Response surfaces of changes in water absorption by mass of testing samples carbonated by dynamic (**1**) and static (**2**) methods, depending on: (**a**) the molding pressure and the concentration of CO_2_; (**b**) the content of lime dust and molding pressure pressing; (**c**) the content of lime dust and the concentration of CO_2_ (at zero level of variation of other factors).

**Figure 10:**
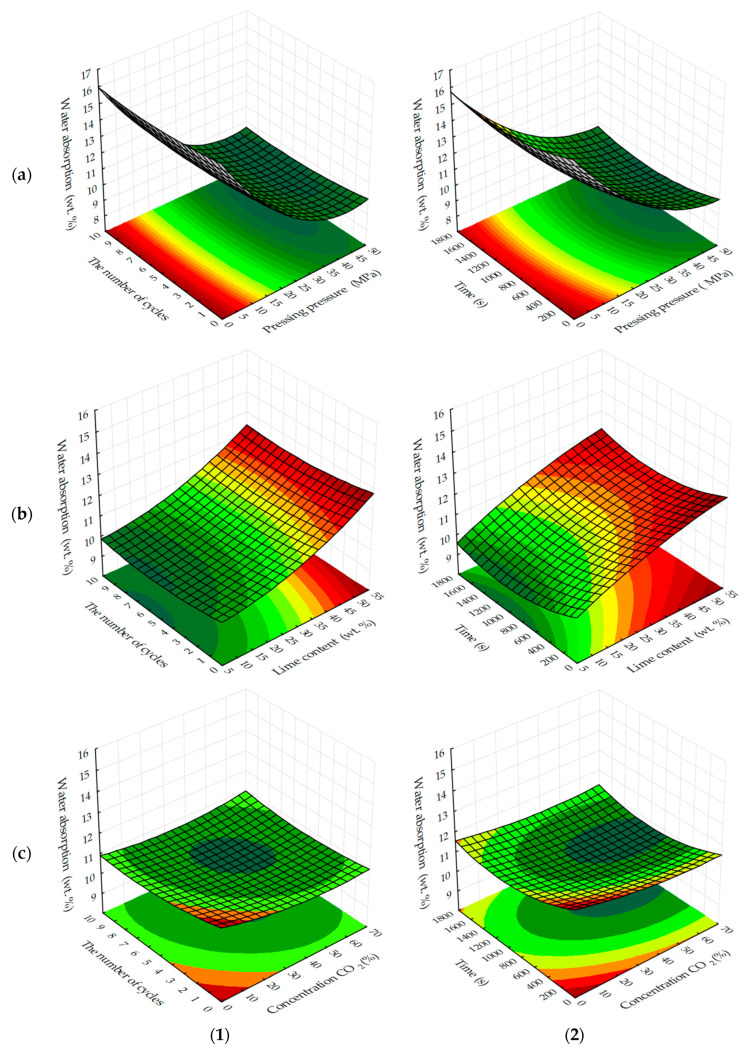
Response surfaces to changes in water absorption by mass of testing samples carbonated by dynamic (**1**) and static (**2**) methods, depending on the number of cycles/carbonation time and: (**a**) pressing pressure; (**b**) lime dust content; (**c**) CO_2_ concentration (at zero level of variation of other factors).

Experimental data on changes in water absorption by weight of testing cylinder samples, based on lime dust and finely dispersed limestone (Figure 9 and Figure 10) show that the minimum achievable values of this parameter, depending on the varying factors in the experiment are in the range from 9% wt. to 10% wt. In contrast to the water resistance, the maximum values of which are achieved with the least effort to form testing samples; the minimum values of water absorption of testing samples, on the contrary, are achieved with the maximum effort of pressing testing samples. 

W_m_ also decreases with a decrease in the content of lime dust in the studied samples, i.e., the most dispersed raw material component, which creates small open pores in the samples, contributing to water migration and water saturation. The change of modes of forced carbonation of testing samples does not change the general logical change of water absorption of samples, but it should be noted that in a static mode under equivalent conditions of variation of factors, W_m_ index is 1%–1.5% less than the mass.

**Figure 11:**
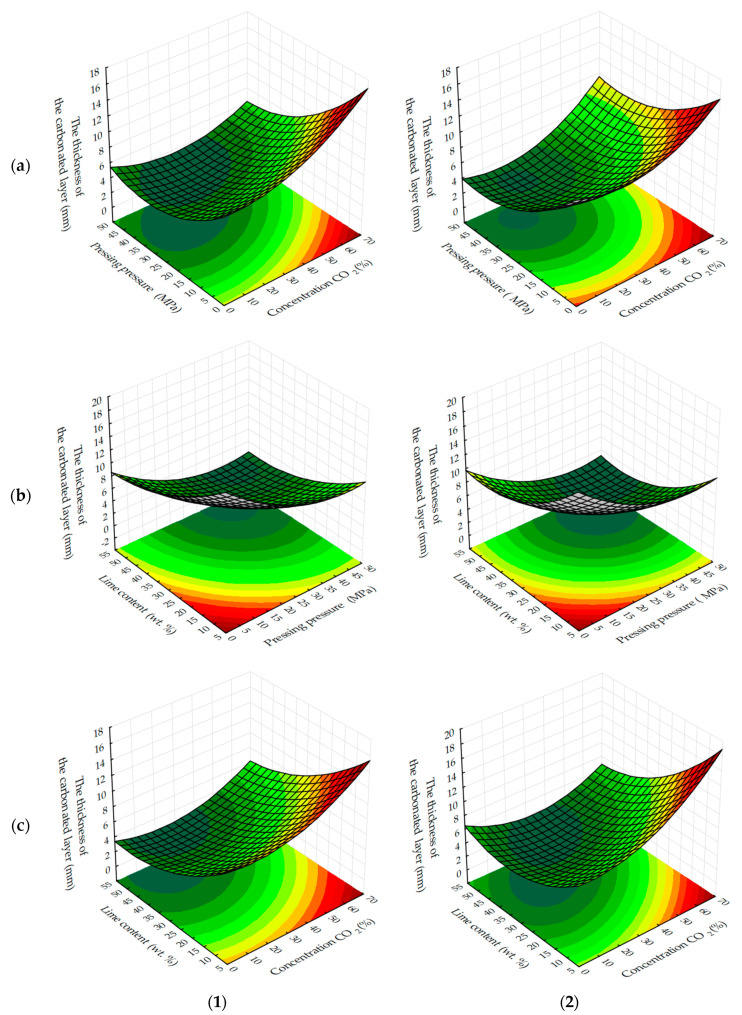
Response surfaces to changes in the thickness of the carbonated layer of testing samples carbonated by dynamic (**1**) and static (**2**) methods, depending on: (**a**) the molding pressure and the concentration of CO_2_; (**b**) the content of lime dust and molding pressure pressing; (**c**) the content of lime dust and the concentration of CO_2_ (at zero level of variation of other factors).

**Figure 12:**
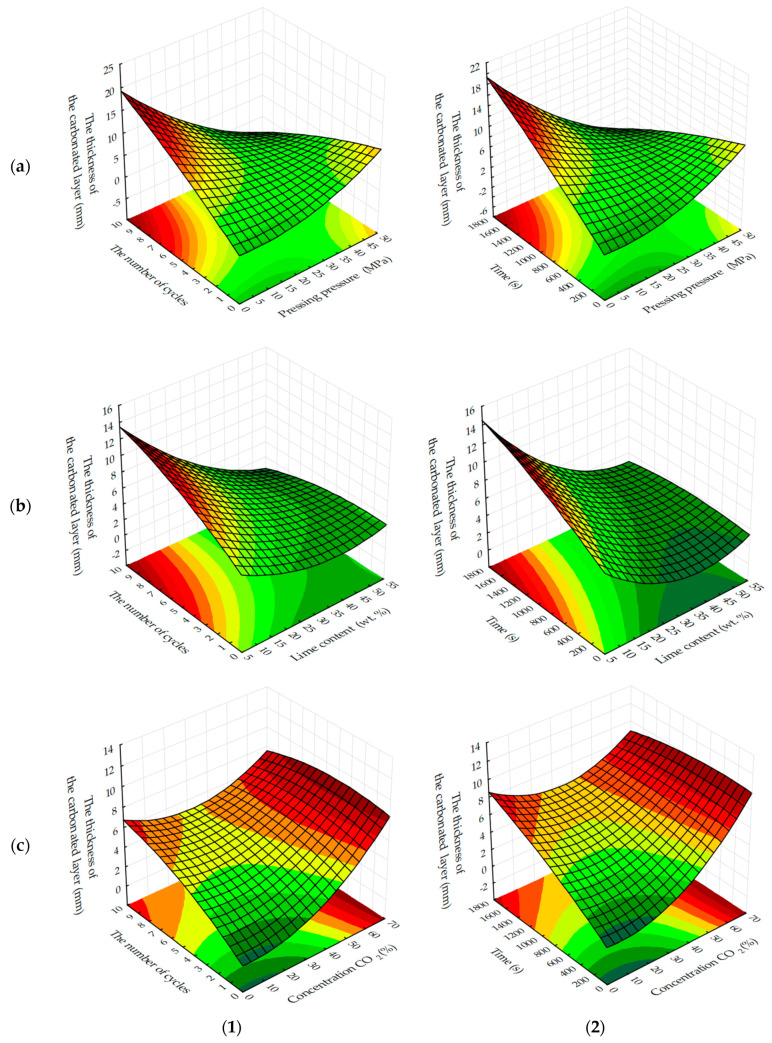
Response surfaces to changes in the thickness of the carbonated layer of samples carbonated by dynamic (**1**) and static (**2**) methods, depending on the number of cycles/time of carbonation and: (**a**) forming pressure of pressing; (**b**) lime dust content; (**c**) concentration of CO_2_ (at zero level of variation of other factors).

**Figure 13:**
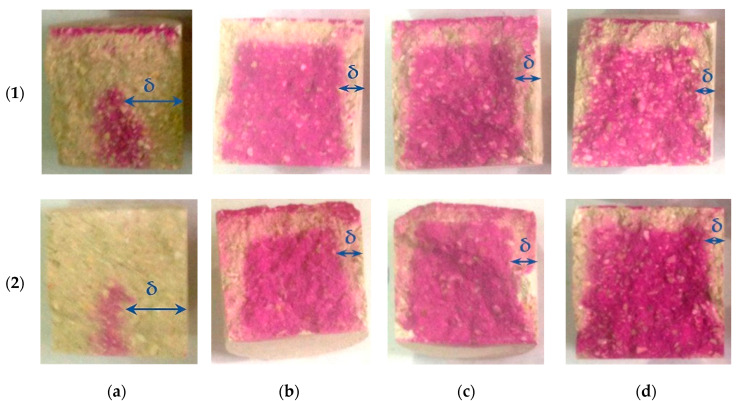
The thickness of the carbonated layer of testing samples obtained under dynamic (**1**) and static (**2**) modes of forced carbonation during the experiment: (**a**) point 7 of the experiment design; (**b**); point 9; (**c**) point 16; (**d**) point 25.

The analysis of response surfaces of the carbonated layer formation of testing samples (Figure 11 and Figure 12) shows that their character of change is very similar to that of water resistance change response surfaces (see Figure 7 and Figure 8). This indicates that the water-resistance of materials hardening due to forced carbonation determines the thickness of the formed carbonated layer, the size of which can indirectly be judged on the degree of carbonation of the lime component in the testing samples (Figure 13). The size of the formed carbonated layer depends on the factors that determine the initial structure of the studied lime-liming samples, the content of lime dust and forming pressure pressing. Thus, with a reduction in lime dust content and pressing forming pressure; i.e., when the least dense initial structure of the material is created, the sample is carbonated almost completely (Figure 13a).

In addition to the lime dust content and pressing forming pressure, the formation of the carbonated layer is largely dependent on the time of forced carbonate processing of sample cylinders and the concentration of CO_2_ in the gas and air mixture fed into the carbonation chamber. As the duration of carbonizing treatment increases and the amount of carbon dioxide in the gas and air mixture increases, the carbonated layer constantly increases (see Figure 12). Thus, absolute values of thickness of the carbonated layer formed in testing samples at a dynamic and static method of their forced carbonation are practically identical, and the main technological factor forming a layer is a time of forced carbonation. Slightly increased δ values observed in testing samples obtained in a static carbonation mode can be attributed to a longer contact time of the samples with CO_2_, due to their continuous maintenance in the CO_2_ gas and air environment, whereas in a dynamic method, due to the cyclic feeding of the gas and air mixture into the carbonation chamber, the total contact time of the samples with CO_2_ was not much, but still less.

Photos in Figure 13 show the carbonated layer formed in the testing samples as a result of their forced carbonation at different modes. 

The carbonated layer was determined on the split cylinder samples by spraying the phenolphthalein alcohol solution to the sample. The visual examination of phenolphthalein-colored sample shows that the formation of carbonate stone, which makes up the carbonated layer, starts from the sample surface, gradually moving inward with the diffusion limitation of carbon dioxide, and the thickness of the layer is not the same in the surface area of the samples. There is practically no carbonated layer in the lower part of the testing samples. This can be explained by the dependence of the Ca(OH)_2_ carbonation process from moisture. It is known [22,25,29,30,31] that at a high humidity of the system chemical reaction of Ca(OH)_2_, carbonation stops. Under the conditions of the experiment, water released as a result of intensive carbonation of the testing samples accumulated in the lower part and prevented the carbonation reaction in this scope of the samples. In this regard, one of the necessary conditions for the production of forced carbonate hardening of products is to ensure an intensive removal of moisture in the carbonation chambers, both from samples and from the chamber itself, for example, "airing" the chamber by a flow of gas and air mixture, with a constant concentration of CO_2_.

A visual analysis of the sample also allowed one to determine that at close values of δ depth of CO_2_ penetration differs depending on the modes of forced carbonation of the cylinder samples. Thus, in samples carbonated by the dynamic method, the inner non-carbonated part after spraying phenolphthalein alcohol solution on it has a pale color in comparison with samples carbonated by the static method. This may indicate a deeper penetration of the carbon dioxide in forced carbonation in a dynamic (cyclic) mode. It should be noted that despite clear boundaries, the carbonated layer does not peel off the uncarbonated part of the sample, representing a solid heterogeneous conglomerate.

## 4. Discussion

As can be seen from the presented experimental calculation results of the study, it is unambiguously difficult to interpret them. It makes sense to consider the process of formation of the basic properties of carbonate hardening materials, depending on the intensity of their forced carbonation more complex, combining the maximum number of factors.

**Figure 14:**
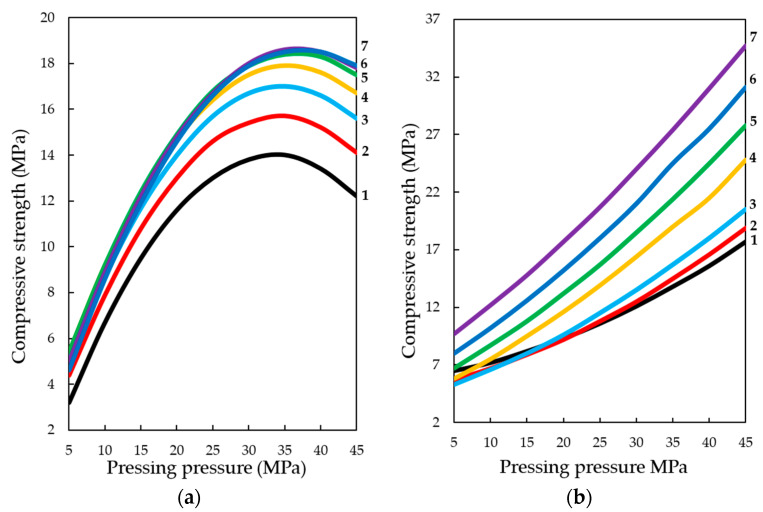
Change in compressive strength of testing samples made from a raw mix containing 22% wt. of lime dust (equivalent to Ca(OH)_2_—10% wt.) carbonated by: (**a**) dynamic and (**b**) static methods, during 5 cycles / 900 s, depending on the forming pressure of pressing and concentration of СО_2_, %: **1**—10; **2**—20; **3**—30; **4**—40; **5**—50; **6**—60; **7**—65.

**Figure 15:**
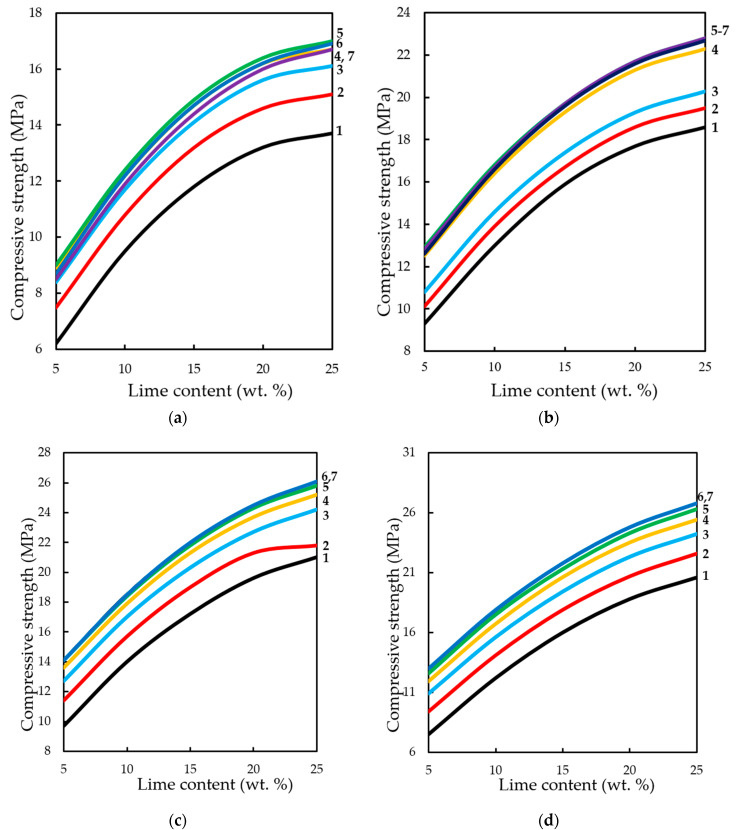
Variation in compressive strength of testing samples manufactured at pressing pressure of: (**a**) 10; (**b**) 20; (**c**) 30; (**d**) 40 MPa. Samples carbonated by dynamic method after five cycles of carbonation, depending on the content of Ca(OH)_2_ and concentration of СО_2_, %: **1—**10; **2—**20; **3**—30; **4—**40; **5**—50; **6**—60; **7**—65.

**Figure 16:**
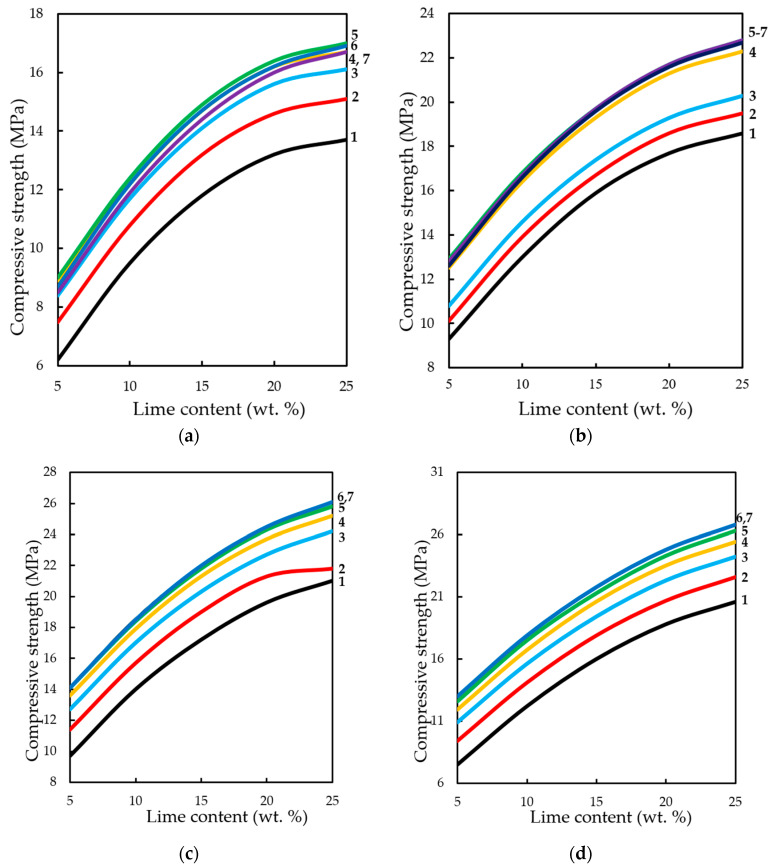
Variation in the compressive strength of testing samples manufactured at pressing pressure of: (**a**) 10; (**b**) 20; (**c**) 30; (**d**) 40 MPa. Samples carbonated by static method during 900 s, depending on the content of Ca(OH)_2_ and concentration of СО_2_, %: **1—**10; **2—**20; **3**—30; **4—**40; **5**—50; **6**—60; **7**—65.

**Figure 17:**
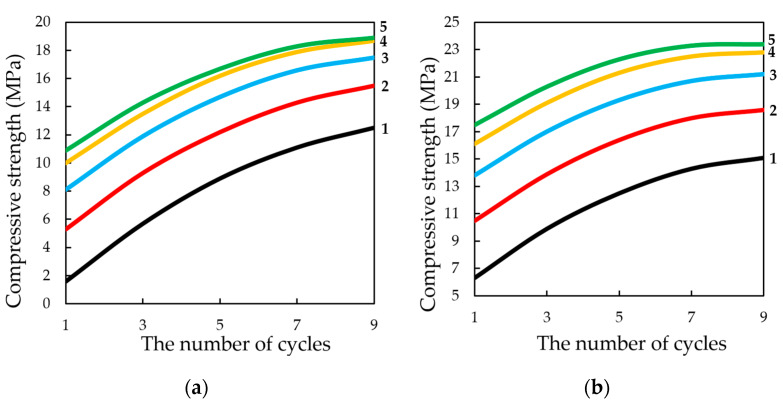

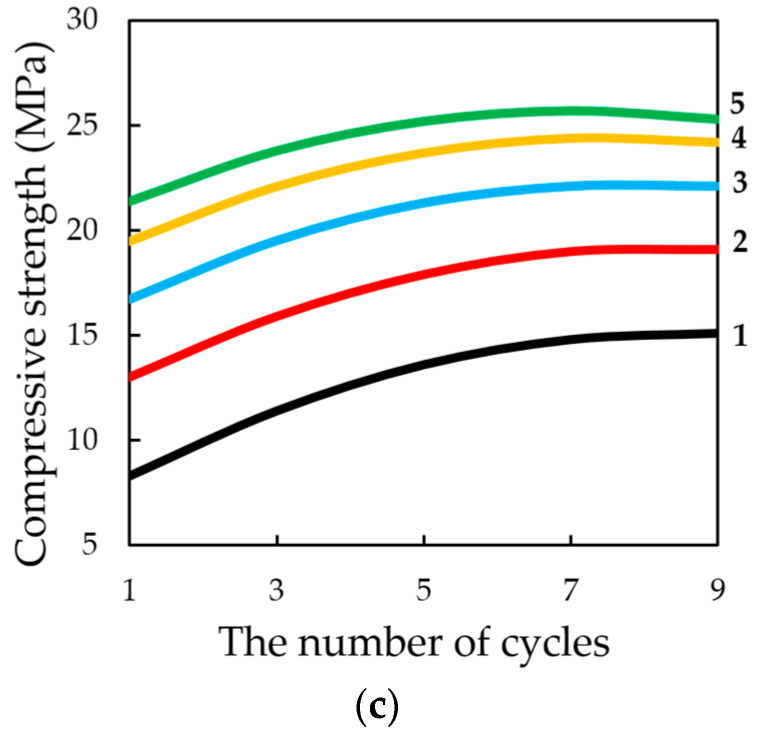
Change in compression strength of testing samples produced by semi-dry pressing at pressing pressure: (**a**) 15; (**b**) 25; (**c**) 35 MPa. Samples carbonated by the dynamic method in gas and air environment with 40% concentration of CO_2_, depending on the number of carbonation cycles and lime content in the raw mix, % wt.: **1**—5; **2**—10; **3**—15; **4**—20; **5**—25.

**Figure 18:**
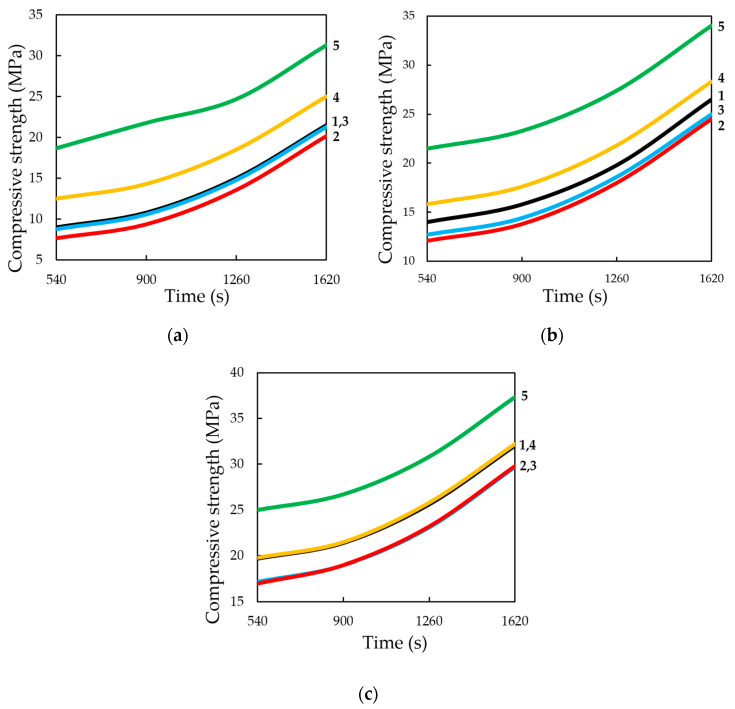
Variation of compressive strength limit of testing samples produced by semi-dry pressing at pressing pressure: (**a**) 15; (**b**) 25; (**c**) 35 MPa. Samples carbonated by the static method in gas and air environment with 40% concentration of CO_2_, depending on carbonation time and lime content in the raw mix, % wt.: **1**—5; **2**—10; **3**—15; **4**—20; **5**—25.

The graphical processing of experimental and calculated data of models of the formation of compressive strength of investigated lime-liming systems on the basis of lime dust, depending on the simultaneous influence of varying factors at various modes of carbonation is presented in Figure 14, Figure 15, Figure 16, Figure 17 and Figure 18.

With the dynamic method of forced carbonate hardening (see Figure 14a), the compressive strength of testing samples increases with an increase in CO_2_ concentration in the gas and air mixture up to 50%; a further increase in CO_2_ concentration up to 65% practically does not affect the change of R_c_. A certain pattern of the dependence of R_c_ testing samples on the forming pressure of the pressing is traced. It can be assumed that with a further increase in the concentration of CO_2_, the compressive strength will be reduced due to the developing corrosion and dissolution of newly formed CaCO_3_ crystals, in accordance with the study of Cizer et al. [29] According to the experimental data, the compressive strength increases with the increase in molding pressure up to 33–38 MPa, after which, with the increase in molding force up to 45 MPa, R_c_ values decrease, which, is explained by a denser structure of the material obtained at such high pressing pressures of the raw mix. Consequently, longer exposure times are required in environments with higher CO_2_ concentrations. Thus, this fact of reduction of R_c_ proves that carbonation component, which forms the future structure of artificial stone from calcite crystals, actively participates in the formation of strength indices of pressed materials, the hardening of which is organized by carbonate type [32,33,34]. 

With the static method of treatment of compositions with carbon dioxide (see Figure 14b), the reverse picture is observed. The dynamics of the strength set of testing samples is lower than in the dynamic method, however, the absolute values of the strength values under similar varying conditions are higher, and the dynamics of the strength set itself tends to further increase, both with increasing forming pressure of pressing samples and with increasing concentration of CO_2_ in the gas and air mixture fed into the carbonation chamber. This phenomenon shows that the acceleration of the lime carbonation reaction by actively seeping CO_2_ through the samples, simulated in the experiment as a dynamic mode of carbonation, firstly, leads to a sharp formation of a large amount of water in the system, which clogs and closes the pores in the material, and complicates the diffusion of CO_2_ in the pores of the material, and secondly, it can create conditions for the emergence in the system of aggressive carbon dioxide and the beginning of the dissolution and corrosion of calcite crystals of the newly formed carbonate structure [23,29,35,36,37].

Estimating the obtained ES samples of forced carbonate hardening of samples on the basis of lime dust and limestone, it can be concluded that the most appropriate way to carry out the accelerated carbonation of materials based on lime, may be a combined method of artificial carbonation, which consists in creating a pre-discharge in the carbonation chamber, the subsequent supply of gas and air mixture of CO_2_ and further conducting the process in a static mode, maintaining a given concentration of CO_2_.

The analysis of changes in compression strength depending on the content of lime binder, forming the pressure of pressing and concentration of CO_2_ in the gas and air mixture at different methods of carbonation, is presented in Figure 15 and Figure 16.

The dynamic method of carbonation (Figure 15) shows that the compressive strength of testing samples increases with increasing binder content, as other factors change. However, as already noted, an increase in CO_2_ concentration of more than 40%–50% of the compressive strength practically does not change, and with the growth of molding pressure of pressing increases the limit concentrations of CO_2_, at which R_c_ practically does not change. Thus, for testing samples manufactured with a pressing force of 10 and 20 MPa, the maximum concentration of CO_2_ is 40%, and for samples pressed at 30 and 40 MPa—60%.

In the conditions of static carbonation (Figure 16) in testing samples with increasing content of Ca(OH)_2_ up to 15% wt. in their composition, there is some decrease of compressive strength, with increasing content of lime in the composition—the strength increases. It seems that this could be an error in program calculation. The strength at the content of calcium hydroxide in the moulding mixtures within 5% wt.–15% wt. changes insignificantly, and with an increase in the content of Ca(OH)_2_ in compositions over 15% wt., the strength increases markedly. It should be noted that the compressive strength of samples carbonated by the static method at increased pressing pressures (hyper-pressing) over 30 MPa is 25%–35% higher than that of similar samples obtained by the dynamic carbonation method. 

The study of the influence of the number of cycles in the dynamic method of carbonation on the change in compression strength of testing samples, presented in Figure 17, shows that, in general, the strength increases with an increase in the number of cycles of carbonation, as well as with an increase in the number of lime binder in the compositions. However, it should be noted that the nature of growth of R_c_ is such that, with an increase in the number of cycles (more than 5) and molding pressure, the pressing growth of compressive strength is somewhat slowed down, due to difficult access from CO_2_ particles to Ca(OH)_2_ in the deep layers of the sample, due to the blocking pores of the material water released during the carbonation reaction Ca(OH)_2_.

Compressive strength of samples at the static method of carbonation (Figure 18) in contrast to the dynamic method, on the contrary, with increasing time of their treatment with carbon dioxide environment, increases to 1620 s, with a lime content of 25% wt. and the forming pressure of pressing 35 MPa is 37.3 MPa, which exceeds the compressive strength of samples obtained under similar conditions, the carbonation of which proceeded in the dynamic conditions of CO_2_ supply more than 45%.

The formation of water-resistance of materials based on lime carbonation curing, as can be seen from the results obtained, depends on the formation of the carbonated layer, the thickening of which is facilitated by increasing the concentration and processing of samples in environments with a high concentration of CO_2_. Increased factors such as molding pressure and lime content in samples, on the contrary, reduce the thickness of the carbonated layer, mainly due to the formation of a more dense initial structure of the material, which prevents the diffusion of CO_2_ [32,38,39,40].

Water absorption by weight of carbonated samples determines the forming pressure of the pressing, with an increase in which the water absorption index decreases. Increasing the concentration of CO_2_ and the number of carbonation cycles also reduces the water absorption of samples and increasing the content of Ca(OH)_2_ in the raw mix increases the Wm indicator of carbonated material, impairing its hydrophysical properties.

**Figure 19:**
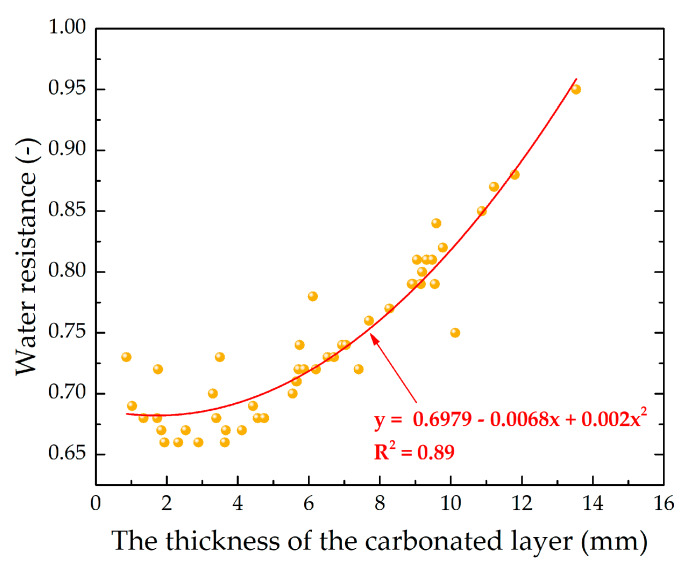
Dependence of water resistance on the thickness of the carbonated layer of lime-liming cylinder samples of semi-dry pressing on the basis of lime dust, obtained at 5 cycles of carbonation in the environment, with 40% concentration of CO_2_.

As was found during the analysis of the research results, the water-resistance of the testing samples depends on the degree of the carbonation of the lime binder, which can be indirectly characterized by the carbonated layer of the samples. Figure 19 shows the graphical dependence of the water-resistance K_s_ on the thickness of the carbonated layer, δ, from which it is seen that the material acquires the properties of water resistance (K_s_ ≥ 0.8) at δ ≥ 9 mm.

In their work, Ergenç et al. [35] showed that the carbonation chamber did not influence the compressive strength of the mortars, but worsened the flexural strength results. Mo et al. [36] subjected cement pastes to CO_2_ under pressure. Their research showed that this resulted in a rapid increase in compressive strength, as well as in microstructure compaction. Ahmad et al. [37] checked the mechanical properties, precisely the compressive strength of the concrete, which was subjected to accelerated carbonation curing. Their research showed that when the concrete was exposed to a pressure of 414 kPa for 10 h, this resulted in an increase in compressive strength. This value increased by 114% compared to the reference sample. In study [41], Moropoulou et al. concluded that mortar with addition of lime powder after 15 months of hardening showed a 236% increase of compressive strength. This is three times the strength increase compared to the reference sample. Chen et al. [42] have observed that carbon curing significantly improves the early strength of cement mortars. However, this positive effect weakens with time. The compressive strength after 3 and 7 days retained initially an increased and then a reduced tendency as the curing time of carbonation was extended. The 28 day strength is slightly dependent on the duration of the carbonation. Based on their research, the authors determined that carbonation curing in combination with a suitable pre-curing can increase the initial compressive strength of the cement mortar. The impact on long term strength is nevertheless limited. Mo et al. [43] in their work carbonated with CO_2_ steel slag paste. The gas was at a pressure of 0.1 MPa. They observed that the creation of CaCO_3_ in pastes reduced the amount of pores and thus improved their compressive strength. Studies carried out by Liu et al. [44] indicated that for steel slag-cement binding materials, the best curing parameters were curing condition at 60 °C and carbonation lasting 7 h. Li et al. [45] tested tricalcium silicate (C_3_S) paste, which is the leading phase in Portland cement. The samples were subjected to accelerated curing with carbon dioxide (4-bar pressure). The results showed that after 2 h of curing, the paste reached a compressive strength of 27.5 MPa and after 24 h up to 62.9 MPa, where the samples subjected to hydration at the same time reached 5.2 MPa and 8.6 MPa, respectively. Cement blended with ground volcanic ash subjected to carbon curing was investigated by Seo et al. [46] They observed a significant increase in compressive strength values. This did not include samples in which cement was replaced in 40% by ground volcanic ash. These samples were considered to be completely carbonated.

In this paper, the thickness of the carbonated layer, δ, was determined by spraying a 1% alcohol solution of phenolphthalein on the cleaved surface of the test sample (see Figure 13). This method allowed only a qualitative analysis of the progress of the chemical reaction of carbonation. In further studies, it is advisable to study the depth of the carbonation layer in the samples, by changing the pH in the direction of the reaction front. In this case, the transition point pH < 9 should be taken as the carbonation depth. As indicated in the study [47], at pH < 9, the brightness of the raspberry color begins to decrease until it disappears. To study the degree of carbonation of the material, it also makes sense to use Thermogravimetric analysis (TGA) in accordance with the work [48].

## 5. Conclusions

As a result of research studies of dynamic and static modes of carbonation of testing, it has been established that the most effective way of the accelerated carbonation hardening of the building materials and products, is a combined method of carbonation. This combination consists of dynamic and static methods. The essence of the combined method is to create a preliminary discharge in the carbonation chamber; the subsequent supply of carbon dioxide gas and air mixture and further carbonation in a static mode, maintaining a constant concentration of CO_2_ in the carbonation chamber is within 30%–40%. One of the necessary conditions for the process of forced carbonation in the manufacturing of building materials and carbonate hardening products is to ensure the intensive removal of moisture by carbonation chambers. Moisture should be removed both from the samples and from the chamber itself, by "aerating" the chamber through the flow of a gas-air mixture with a constant CO_2_ concentration.

The results of the present research studies have shown that the application of the accelerated methods of carbonation of materials on the basis of a binding component containing Ca(OH)_2_, is not effective at high concentration of carbon dioxide in a carbonation chamber (more than 50%). Optimal concentrations of CO_2_ in gas and air mixtures used to produce building materials and forced carbonate hardening products can be considered to be 30%–40%. Approximately, this concentration of carbon dioxide is found, for example, in the kiln and flue gases of coal-fired charcoal kilns and thermal power plants. Taking into account the fact that at high concentrations of CO_2_ defects develop in the structure of forced carbonated material, it is more appropriate to increase the carbonation time.

## Figures and Tables

**Table 1:** Changes in the content of the main phases in lime dust under various conditions of its preliminary preparation (% wt).

Lime Dust Preparation Conditions	Са(ОН)_2_ (eq. to СаО)	СаСО_3_
In its natural condition	40.3 (30.5)	32.5
After quenching	50.6 (38.3)	32.5
Slaked and tempered for 120 min	50.9 (38.5)	32.6
Slaked and tempered for 420 min	55.1 (41.7)	33.0
Slaked and tempered for 420 min with additional boiling	59.5 (45.0)	34.0

**Table 2:** Characteristics of Balaklava deposit limestone.

Density	Average Density	Porosity	Grade according to Crushability	CaCO_3_ Content
(g·cm^−3^)	(g·cm^−3^)	(%)	(-)	(%)
2.73	2.41	11.7	1000	94.8

**Table 3:** Conditions of the experiment.

Factor	Units	Levels of Variation				
		−2	−1	0	1	2
z_1_	%	5	20	35	50	65
z_2_	МPа	5	15	25	35	45
z_3_	% wt.	5	10	15	20	25
z_4_	the number of cycles/time, s	1/180	3/540	5/900	7/1260	9/1620

**Table 4:** Properties of testing samples after carbonation in dynamic (1) and static (2) ways.

No.	Factor				Optimized Parameters									
					R_c_, МPa		*ρ*_o_, g·cm^−3^		K_s_		W_m_, %		δ, mm	
	z_1_	z_2_	z_3_	z_4_	(1)	(2)	(1)	(2)	(1)	(2)	(1)	(2)	(1)	(2)
1	1	1	1	1	27.36	34.47	2.101	2.050	0.70	0.71	10.10	9.91	1.33	3.00
2	−1	1	1	1	19.81	15.40	2.063	2.064	0.65	0.70	10.44	10.53	2.67	3.33
3	1	−1	1	1	19.44	19.47	1.976	1.978	0.80	0.75	12.78	12.68	5.33	6.00
4	−1	−1	1	1	15.76	13.20	1.983	1.953	0.86	0.89	12.34	12.43	6.67	7.67
5	1	1	−1	1	20.29	26.17	2.174	2.211	0.87	0.70	9.01	9.20	6.00	7.67
6	−1	1	−1	1	15.66	16.73	2.142	2.150	0.72	0.70	9.44	9.34	3.00	3.67
7	1	−1	−1	1	13.83	14.03	2.095	2.104	0.85	0.85	11.71	11.80	12.00	12.33
8	−1	−1	−1	1	13.40	13.77	2.023	2.024	0.82	0.81	11.31	11.29	10.33	11.00
9	1	1	1	−1	21.80	19.87	2.066	2.018	0.59	0.62	10.56	10.66	4.00	4.17
10	−1	1	1	−1	23.50	22.77	2.065	2.066	0.59	0.60	10.18	10.22	2.33	2.83
11	1	−1	1	−1	14.34	15.33	1.966	1.952	0.73	0.68	12.50	12.68	6.67	6.67
12	−1	−1	1	−1	14.44	15.23	1.957	1.954	0.55	0.55	12.82	12.72	2.33	2.67
13	1	1	−1	−1	16.52	21.67	2.132	2.137	0.73	0.75	9.12	9.38	5.67	8.00
14	−1	1	−1	−1	15.66	17.57	2.149	2.154	0.63	0.63	9.48	9.34	7.00	6.00
15	1	−1	−1	−1	11.32	11.77	2.052	2.050	0.63	0.63	11.53	11.52	6.67	7.17
16	−1	−1	−1	−1	8.59	4.53	2.080	1.959	0.77	0.63	11.65	12.27	4.67	4.83
17	2	0	0	0	18.21	18.47	2.098	2.088	0.82	0.80	10.59	10.51	9.67	9.50
18	−2	0	0	0	13.49	10.67	2.083	2.120	0.59	0.65	11.19	11.77	3.00	3.33
19	0	2	0	0	19.06	18.23	2.170	2.149	0.72	0.75	9.06	9.13	3.33	2.67
20	0	−2	0	0	5.47	7.17	1.827	1.907	0.79	0.73	15.05	14.81	9.67	9.33
21	0	0	2	0	19.91	20.20	1.987	1.988	0.65	0.65	11.96	11.74	2.67	3.17
22	0	0	−2	0	11.51	12.37	2.144	2.154	0.76	0.75	10.00	9.80	9.33	9.17
23	0	0	0	2	20.19	22.73	2.077	2.074	0.58	0.71	10.63	11.17	5.33	5.17
24	0	0	0	−2	11.32	9.27	2.043	1.960	0.67	0.58	11.03	11.60	3.00	2.67
25	0	0	0	0	19.16	13.67	2.063	2.034	0.63	0.67	10.42	11.00	3.33	4.00
26	0	0	0	0	19.96	12.67	2.078	2.052	0.72	0.68	10.55	10.74	4.67	4.83
27	0	0	0	0	18.68	13.60	2.072	2.047	0.72	0.64	10.31	10.50	4.33	4.67
28	0	0	0	0	19.01	13.73	2.073	2.051	0.65	0.64	10.39	10.55	4.33	4.17
29	0	0	0	0	18.85	13.63	2.075	2.023	0.69	0.65	10.45	10.71	4.33	4.17
30	0	0	0	0	18.95	13.37	2.065	2.047	0.68	0.63	10.31	10.47	3.33	3.67
31	0	0	0	0	19.20	13.63	2.075	2.049	0.69	0.66	10.34	10.53	4.00	4.33

**Table 5:** Correlation coefficients of ES models of changes in basic properties of testing samples obtained by dynamic (1) and static (2) carbonation methods.

Indication of Coefficients	Coefficients of ES Models of the Investigated Parameters									
	R_c_, МPa		*ρ*_o_, g·cm^−3^		K_s_		W_m_, %		δ, mm	
	(1)	(2)	(1)	(2)	(1)	(2)	(1)	(2)	(1)	(2)
b_o_	18.97	23.04	2.072	2.043	0.68	0.66	9.97	10.64	4.05	4.26
b_1_	1.15	2.47	0.005	±0	0.03	0.02	−0.06	−0.12	0.92	1.06
b_2_	3.195	3.73	0.060	0.057	−0.03	−0.01	−1.26	−1.26	−1.47	−1.38
b_3_	2.42	1.88	−0.041	−0.045	−0.03	−0.02	0.52	0.48	−1.56	−1.51
b_4_	1.55	2.14	0.007	0.020	0.04	0.05	−0.06	−0.10	0.53	0.72
b_11_	−0.43	0.84	0.006	0.015	0.01	0.02	−0.19	0.07	0.52	0.61
b_22_	−1.33	0.37	−0.017	±0	0.02	0.02	−0.48	0.28	0.56	0.51
b_33_	−0.47	1.27	±0	±0	0.01	0.01	0.21	±0	0.43	0.55
b_44_	−0.46	1.2	±0	−0.007	−0.01	±0	0.17	0.13	±0	±0
b_12_	0.29	0.99	±0	−0.013	0.02	0.01	−0.07	±0	−0.29	±0
b_13_	0.05	±0	±0	−0.016	±0	−0.01	0.04	±0	±0	±0
b_14_	0.91	1.66	0.011	±0	±0	−0.02	0.03	±0	−0.29	±0
b_23_	0.47	−0.55	0.004	−0.010	−0.02	−0.01	±0	±0	±0	±0
b_24_	−0.51	±0	−0.003	±0	−0.02	−0.04	±0	±0	−1.25	−1.19
b_34_	−0.18	±0	0.003	±0	±0	0.01	±0	±0	−0.42	±0
