# Correction: Lyubomirskiy, N., et al. Intensive Ways of Producing Carbonate Curing Building Materials Based on Lime Secondary Raw Materials. Materials 2020, Vol. 13, 2304

**DOI:** 10.3390/ma13163477

**Published:** 2020-08-07

**Authors:** Nikolai Lyubomirskiy, Aleksandr Bakhtin, Stanisław Fic, Małgorzata Szafraniec, Tamara Bakhtinа

**Affiliations:** 1 Department of Civil Engineering and Materials Science, Faculty of Architecture and Civil Engineering, Academy of Civil Engineering and Architecture, V.I. Vernadsky Crimean Federal University, Prospekt Vernadskogo 4, 295007 Simferopol, Republic of Crimea; niklub.ua@gmail.com (N.L.); aleserba@gmail.com (A.B.); t.bakhtina83@gmail.com (T.B.); 2 Faculty of Civil Engineering and Architecture, Lublin University of Technology, ul. Nadbystrzycka 40, 20-618 Lublin, Poland; s.fic@pollub.pl

The authors wish to make the following correction to this paper [1]. Due to duplication of the same diagrams in Figures 15 and 16, replace:


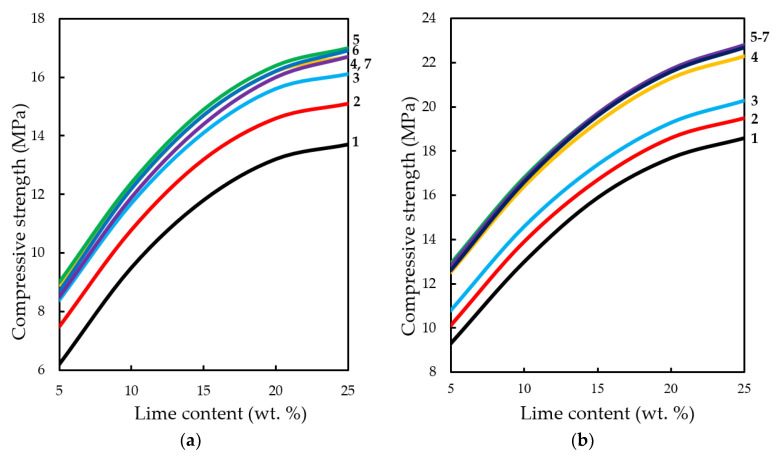



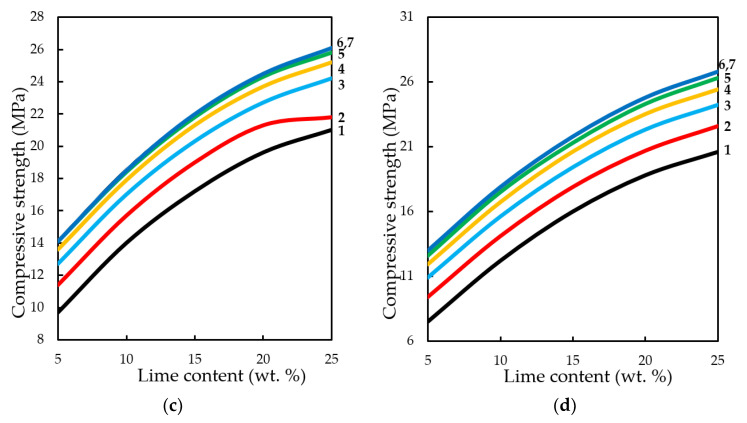

**Figure 16.** Variation in the compressive strength of testing samples manufactured at pressing pressure of: (**a**) 10; (**b**) 20; (**c**) 30; (**d**) 40 MPa. Samples carbonated by static method during 900 s, depending on the content of Ca(OH)_2_ and concentration of СО_2_, %: **1**—10; **2**—20; **3**—30; **4**—40; **5**—50; **6**—60; **7**—65.

with


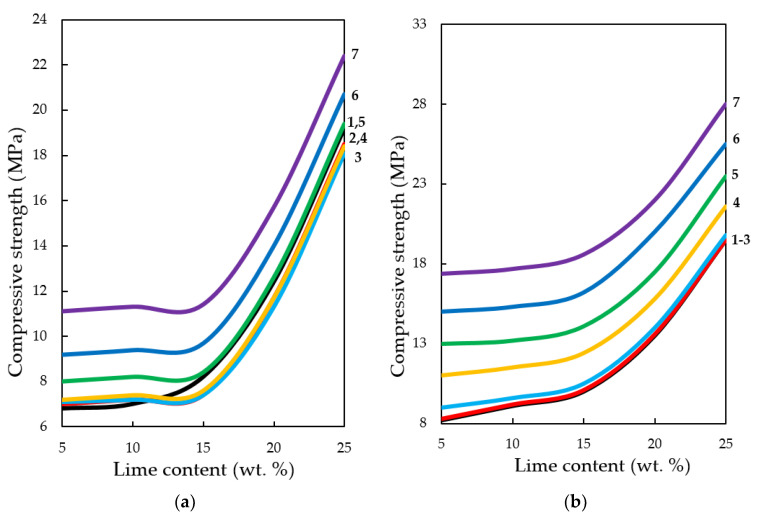



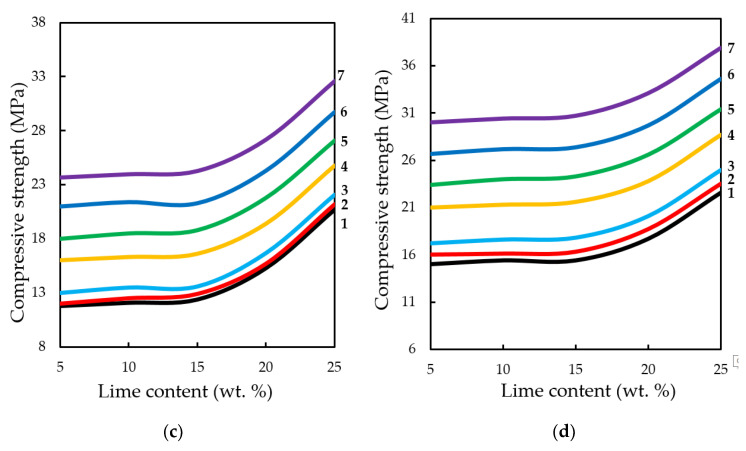

**Figure 16.** Variation in the compressive strength of testing samples manufactured at pressing pressure of: (**a**) 10; (**b**) 20; (**c**) 30; (**d**) 40 MPa. Samples carbonated by static method during 900 s, depending on the content of Ca(OH)_2_ and concentration of СО_2_, %: **1**—10; **2**—20; **3**—30; **4**—40; **5**—50; **6**—60; **7**—65.

The authors would like to apologize for any inconvenience caused to the readers by these changes.

## References

[1] Lyubomirskiy N., Bakhtin A., Fic S., Szafraniec M., Bakhtina T. (2020). Intensive ways of producing carbonate curing building materials based on lime secondary raw materials. Materials (Basel).

